# (*E*)-1-[(3-Iodo­phen­yl)imino­meth­yl]naphthalen-2-ol

**DOI:** 10.1107/S1600536812036793

**Published:** 2012-09-29

**Authors:** Tufan Akbal, Ayşen Ağar Alaman, Sümeyye Gümüş, Ahmet Erdönmez

**Affiliations:** a Ondokuz Mayıs University, Arts and Sciences Faculty, Department of Physics, 55139 Samsun, Turkey; b Ondokuz Mayıs University, Arts and Sciences Faculty, Department of Chemistry, 55139 Samsun, Turkey

## Abstract

In the title mol­ecule, C_17_H_12_INO, the dihedral angle between the naphthaldeyde plane and the 3-iodo­aniline plane is20.07 (13)°. It exists in the solid state as an enol–imine tautomer with a strong intra­molecular O—H⋯N hydrogen bond.

## Related literature

For the applications of iodoaromatic compounds in synthetic organic chemistry, medicine and biochemistry, see; Merkushev (1988 ▶); Olah *et al.* (1993 ▶). Schiff base complexes have been used in catalytic reactions and are used as models for biological systems, see: Hamilton *et al.* (1987 ▶); Pyrz *et al.* (1985 ▶); Costamagna *et al.* (1992 ▶). For related structures, see: Ünver *et al.* (2000 ▶); Manvizhi *et al.* (2011 ▶).
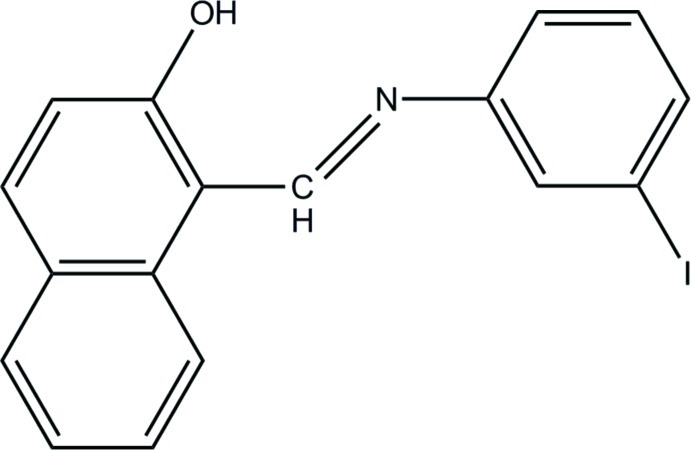


## Experimental

### 

#### Crystal data

C_17_H_12_INO
*M* *_r_* = 373.18
Monoclinic, 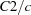

*a* = 32.059 (3) Å
*b* = 4.8392 (3) Å
*c* = 19.2682 (16) Å
β = 107.269 (6)°
*V* = 2854.5 (4) Å^3^
*Z* = 8
Mo *K*α radiation
μ = 2.24 mm^−1^
*T* = 296 K
0.80 × 0.30 × 0.03 mm

#### Data collection

Stoe IPDS 2 diffractometer
Absorption correction: integration (*X-RED32*; Stoe & Cie, 2002 ▶) *T*_min_ = 0.793, *T*_max_ = 0.925
9569 measured reflections
2781 independent reflections
1607 reflections with *I* > 2σ(*I*)
*R*_int_ = 0.056

#### Refinement

*R*[*F* ^2^ > 2σ(*F* ^2^)] = 0.042
*wR*(*F* ^2^) = 0.094
*S* = 0.93
2781 reflections
181 parameters
H-atom parameters constrained
Δρ_max_ = 0.87 e Å^−3^
Δρ_min_ = −0.57 e Å^−3^

### 

Data collection: *X-AREA* (Stoe & Cie, 2002 ▶); cell refinement: *X-AREA*; data reduction: *X-RED32* (Stoe & Cie, 2002 ▶); program(s) used to solve structure: *WinGX* (Farrugia, 1997 ▶) and *SHELXS97* (Sheldrick, 2008 ▶); program(s) used to refine structure: *SHELXL97* (Sheldrick, 2008 ▶); molecular graphics: *ORTEP-3* for Windows (Farrugia, 1997 ▶); software used to prepare material for publication: *WinGX* (Farrugia, 1999 ▶) and *PLATON* (Spek, 2009).

## Supplementary Material

Crystal structure: contains datablock(s) I, global. DOI: 10.1107/S1600536812036793/zj2093sup1.cif

Structure factors: contains datablock(s) I. DOI: 10.1107/S1600536812036793/zj2093Isup2.hkl

Supplementary material file. DOI: 10.1107/S1600536812036793/zj2093Isup3.mol

Supplementary material file. DOI: 10.1107/S1600536812036793/zj2093Isup4.cml

Additional supplementary materials:  crystallographic information; 3D view; checkCIF report

## Figures and Tables

**Table 1:** Hydrogen-bond geometry (Å, °)

*D*—H⋯*A*	*D*—H	H⋯*A*	*D*⋯*A*	*D*—H⋯*A*
O1—H1⋯N1	0.82	1.82	2.555 (6)	148

**Fig. 1.**
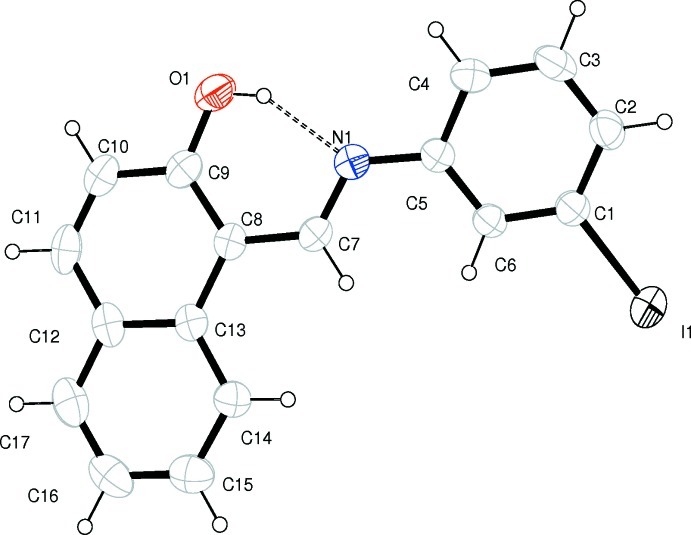
The title molecule with the atom-numbering scheme. The displacement ellipsoids are drawn at the 30% probability level. The dashed line indicates the intramolecular hydrogen bond.

**Fig. 2.**
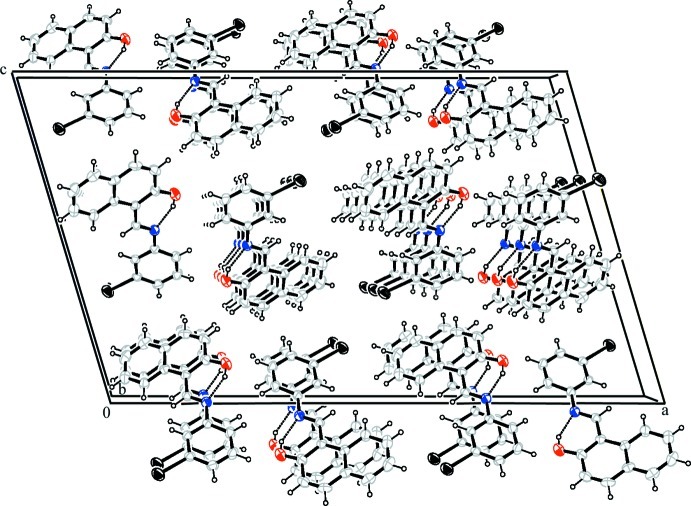
An ORTEP-3 (Farrugia, 1997) packing diagram of (I), viewed along the b axis.

## supplementary crystallographic information

### Comment

Iodoaromatic compounds are valuable and versatile synthetic intermediates in many domains of synthetic organic chemistry, medicine and biochemistry (Merkushev *et al.*, 1988; Olah *et al.*, 1993). The Schiff base complexes have also been used in catalytic reactions (Hamilton *et al.*, 1987) are used as models for biological systems (Pyrz *et al.*, 1985; Costamagna *et al.*, 1992). There are two types of intramolecular hydrogen bonds in Schiff bases, namely keto-amine (N—H···O) and enol-imine (N···H—O) tautomeric forms. The present X-ray investigation shows that the title compound,(I), prefers the enol-imine tautomeric form rather than the keto-amine tautomeric form. The C9—O1 and C7—N1 bond lengths verify the enol-imine tautomeric form. these distances agree with the literature[1.310 (8) and 1.319 (6)Â; Ünver *et al.* 2000], which also show the enol-imine tautomeric form. The C1—I1 bond lenght in (I) is also in a good agremeent with the corresponding distances in the literature [2.092 (4)Â; Manvizhi *et al.*, 2011]. The bond distances for O1-H1O1 and N1-H1O1 are 0.82 and 1.82 Â, respectively, and the N1···H1—O1 angle is 148 Å. These distances and angle agree with the literature[Ünver *et al.* 2000], The title molecule with the atom-numbering scheme. The displacement ellipsoids are drawn at the 30% probability level. The dashed line indicates the intramolecular hydrogen bond. An *ORTEP-3* (Farrugia, 1997) packing diagram of (I), viewed along the *b* axis. The molecule is non-planar. The angle between the two Schiff base moieties [C1—C6,N1,I1] and [C7—C13,O1,N1] is 20.07 (13) Å. *Cg*(1), *Cg*(2) and *Cg*(3) are the centroids of rings C1—C6, C8—C13 and C12—C17, respectively. However, π···π interactions between the centroids of the *Cg*(1) and *Cg*(2) rings (distance between ring centroids = 4.664 (3) Â), and the *Cg*(2) and *Cg*(3) rings (distance between ring centroids = 4.791 (3)Â), stack the molecules along the *b*-axis.

### Experimental

The compound *E*)—*1*-((3-iodophenyllimino)methyl)naphthalen-2-ol (*E*)-1-((3-bromophenyllimino)methyl)naphthalen-2-ol was prepared by refluxing a mixture of a solution containing 2-hydroxy-1-naphthaldehyde (17.2 mg 0.100 mmol) in 30 ml absolute ethanol and a solution containing 3-iodoaniline (21.9 mg 0.100 mmol) in 20 ml absolute ethanol. The reaction mixture was stirred for 4 h under reflux. Single crystals of the title compound for X-ray anaysis were obtaned by slow evaporation of an ethaol solition (yield % 67; m.p 410–412 *^o^*K).

### Refinement

All carbon attached H-atoms were refined using riding model for hydrogen bonds with d(C—H) = 0.93 Å (*U*_iso_=1.2*U*_eq_ of the parent atom) for aromatic carbon atoms and d(C—H) = 0.96 Å (*U*_iso_=1.5*U*_eq_ of the parent atom) for methyl carbon atoms.

### Crystal data

**Table:**

C_17_H_12_INO	*F*(000) = 1456
*M**_r_* = 373.18	*D*_x_ = 1.737 Mg m^−^^3^
Monoclinic, *C*2/*c*	Melting point = 410–412 K
Hall symbol: -C 2yc	Mo *K*α radiation, λ = 0.71073 Å
*a* = 32.059 (3) Å	Cell parameters from 9569 reflections
*b* = 4.8392 (3) Å	θ = 1.3–26.0°
*c* = 19.2682 (16) Å	µ = 2.24 mm^−^^1^
β = 107.269 (6)°	*T* = 296 K
*V* = 2854.5 (4) Å^3^	Needle, yellow
*Z* = 8	0.80 × 0.30 × 0.03 mm

### Data collection

**Table:**

Stoe IPDS 2 diffractometer	2781 independent reflections
Radiation source: fine-focus sealed tube	1607 reflections with *I* > 2σ(*I*)
Graphite monochromator	*R*_int_ = 0.056
w–scan rotation	θ_max_ = 26.0°, θ_min_ = 1.3°
Absorption correction: integration (*X-RED32*; Stoe & Cie, 2002)	*h* = −38→38
*T*_min_ = 0.793, *T*_max_ = 0.925	*k* = −5→5
9569 measured reflections	*l* = −23→23

### Refinement

**Table:**

Refinement on *F*^2^	Secondary atom site location: difference Fourier map
Least-squares matrix: full	Hydrogen site location: inferred from neighbouring sites
*R*[*F*^2^ > 2σ(*F*^2^)] = 0.042	H-atom parameters constrained
*wR*(*F*^2^) = 0.094	*w* = 1/[σ^2^(*F*_o_^2^) + (0.0405*P*)^2^] where *P* = (*F*_o_^2^ + 2*F*_c_^2^)/3
*S* = 0.93	(Δ/σ)_max_ = 0.001
2781 reflections	Δρ_max_ = 0.87 e Å^−^^3^
181 parameters	Δρ_min_ = −0.57 e Å^−^^3^
0 restraints	Extinction correction: *SHELXL97* (Sheldrick, 2008)
Primary atom site location: structure-invariant direct methods	Extinction coefficient: 0

### Special details

**Table:**

Geometry. All e.s.d.'s (except the e.s.d. in the dihedral angle between two l.s. planes) are estimated using the full covariance matrix. The cell e.s.d.'s are taken into account individually in the estimation of e.s.d.'s in distances, angles and torsion angles; correlations between e.s.d.'s in cell parameters are only used when they are defined by crystal symmetry. An approximate (isotropic) treatment of cell e.s.d.'s is used for estimating e.s.d.'s involving l.s. planes.
Refinement. Refinement of *F*^2^ against ALL reflections. The weighted *R*-factor *wR* and goodness of fit *S* are based on *F*^2^, conventional *R*-factors *R* are based on *F*, with *F* set to zero for negative *F*^2^. The threshold expression of *F*^2^ > σ(*F*^2^) is used only for calculating *R*-factors(gt) *etc*. and is not relevant to the choice of reflections for refinement. *R*-factors based on *F*^2^ are statistically about twice as large as those based on *F*, and *R*- factors based on ALL data will be even larger.

### Fractional atomic coordinates and isotropic or equivalent isotropic displacement parameters (Å^2^)

**Table:**

	*x*	*y*	*z*	*U*_iso_*/*U*_eq_	
I1	0.052729 (12)	0.52255 (9)	0.33319 (2)	0.08465 (19)	
C5	0.16216 (15)	0.0503 (9)	0.4653 (2)	0.0490 (11)	
C8	0.15014 (14)	−0.4836 (10)	0.5955 (2)	0.0498 (11)	
O1	0.22667 (12)	−0.3804 (8)	0.6294 (2)	0.0784 (11)	
H1	0.2177	−0.2766	0.5946	0.118*	
C9	0.19336 (16)	−0.5200 (11)	0.6398 (3)	0.0593 (13)	
C11	0.1710 (2)	−0.8587 (12)	0.7108 (3)	0.0712 (16)	
H11	0.1781	−0.9838	0.7491	0.085*	
N1	0.17155 (13)	−0.1458 (9)	0.5220 (2)	0.0546 (10)	
C4	0.19651 (17)	0.1402 (12)	0.4410 (3)	0.0620 (13)	
H4	0.2243	0.0672	0.4616	0.074*	
C6	0.12123 (16)	0.1594 (10)	0.4331 (2)	0.0514 (12)	
H6	0.0976	0.1010	0.4481	0.062*	
C7	0.14134 (16)	−0.2876 (10)	0.5375 (2)	0.0508 (12)	
H7	0.1125	−0.2607	0.5096	0.061*	
C3	0.19005 (19)	0.3351 (13)	0.3873 (3)	0.0721 (16)	
H3	0.2135	0.3935	0.3720	0.087*	
C2	0.14946 (18)	0.4450 (11)	0.3557 (3)	0.0655 (14)	
H2	0.1451	0.5779	0.3194	0.079*	
C1	0.11518 (16)	0.3532 (11)	0.3793 (2)	0.0544 (12)	
C13	0.11570 (16)	−0.6479 (10)	0.6093 (2)	0.0510 (12)	
C17	0.0936 (2)	−0.9958 (12)	0.6815 (3)	0.0797 (16)	
H17	0.1007	−1.1192	0.7203	0.096*	
C14	0.07180 (17)	−0.6311 (12)	0.5668 (3)	0.0651 (14)	
H14	0.0638	−0.5092	0.5277	0.078*	
C16	0.0511 (2)	−0.9750 (14)	0.6391 (4)	0.0868 (18)	
H16	0.0296	−1.0836	0.6488	0.104*	
C10	0.20262 (19)	−0.7080 (12)	0.6980 (3)	0.0693 (15)	
H10	0.2312	−0.7273	0.7279	0.083*	
C12	0.1268 (2)	−0.8351 (11)	0.6679 (3)	0.0613 (14)	
C15	0.0407 (2)	−0.7938 (14)	0.5825 (4)	0.0810 (17)	
H15	0.0118	−0.7799	0.5538	0.097*	

### Atomic displacement parameters (Å^2^)

**Table:**

	*U*^11^	*U*^22^	*U*^33^	*U*^12^	*U*^13^	*U*^23^
I1	0.0709 (2)	0.0937 (3)	0.0771 (3)	0.0026 (2)	0.00310 (19)	0.0215 (2)
C5	0.053 (3)	0.047 (3)	0.047 (3)	−0.006 (2)	0.014 (2)	−0.009 (2)
C8	0.058 (3)	0.048 (3)	0.045 (2)	0.007 (2)	0.017 (2)	−0.005 (2)
O1	0.054 (2)	0.094 (3)	0.079 (3)	0.005 (2)	0.007 (2)	0.005 (2)
C9	0.058 (3)	0.060 (3)	0.058 (3)	0.010 (3)	0.015 (2)	−0.010 (3)
C11	0.104 (5)	0.060 (4)	0.045 (3)	0.021 (3)	0.014 (3)	0.006 (3)
N1	0.053 (2)	0.055 (3)	0.056 (2)	0.001 (2)	0.016 (2)	−0.005 (2)
C4	0.053 (3)	0.067 (4)	0.067 (3)	−0.011 (3)	0.019 (3)	−0.009 (3)
C6	0.054 (3)	0.056 (3)	0.045 (3)	−0.010 (2)	0.015 (2)	−0.005 (2)
C7	0.050 (3)	0.053 (3)	0.046 (3)	0.004 (2)	0.010 (2)	−0.007 (2)
C3	0.069 (4)	0.085 (4)	0.070 (4)	−0.027 (3)	0.032 (3)	−0.004 (3)
C2	0.073 (3)	0.073 (4)	0.051 (3)	−0.012 (3)	0.018 (3)	0.004 (3)
C1	0.058 (3)	0.060 (3)	0.043 (3)	−0.009 (2)	0.011 (2)	−0.007 (2)
C13	0.060 (3)	0.045 (3)	0.052 (3)	0.005 (2)	0.022 (2)	−0.007 (2)
C17	0.114 (5)	0.059 (4)	0.080 (4)	0.007 (4)	0.049 (4)	0.005 (3)
C14	0.059 (3)	0.068 (4)	0.070 (3)	−0.002 (3)	0.022 (3)	0.001 (3)
C16	0.099 (5)	0.073 (4)	0.107 (5)	−0.017 (4)	0.059 (4)	−0.005 (4)
C10	0.067 (4)	0.071 (4)	0.060 (3)	0.014 (3)	0.004 (3)	0.001 (3)
C12	0.090 (4)	0.048 (3)	0.053 (3)	0.008 (3)	0.032 (3)	−0.001 (2)
C15	0.069 (4)	0.082 (5)	0.097 (5)	−0.006 (3)	0.032 (4)	−0.013 (4)

### Geometric parameters (Å, º)

**Table:**

I1—C1	2.101 (5)	C6—H6	0.9300
C5—C6	1.379 (6)	C7—H7	0.9300
C5—C4	1.387 (6)	C3—C2	1.370 (7)
C5—N1	1.411 (6)	C3—H3	0.9300
C8—C9	1.406 (6)	C2—C1	1.381 (7)
C8—C7	1.428 (6)	C2—H2	0.9300
C8—C13	1.448 (7)	C13—C14	1.406 (7)
O1—C9	1.328 (6)	C13—C12	1.408 (7)
O1—H1	0.8200	C17—C16	1.368 (9)
C9—C10	1.405 (7)	C17—C12	1.404 (8)
C11—C10	1.331 (8)	C17—H17	0.9300
C11—C12	1.416 (7)	C14—C15	1.373 (8)
C11—H11	0.9300	C14—H14	0.9300
N1—C7	1.292 (6)	C16—C15	1.361 (9)
C4—C3	1.370 (8)	C16—H16	0.9300
C4—H4	0.9300	C10—H10	0.9300
C6—C1	1.369 (7)	C15—H15	0.9300
			
C6—C5—C4	118.3 (5)	C3—C2—H2	120.9
C6—C5—N1	124.1 (4)	C1—C2—H2	120.9
C4—C5—N1	117.6 (4)	C6—C1—C2	121.4 (5)
C9—C8—C7	119.3 (4)	C6—C1—I1	119.4 (4)
C9—C8—C13	119.1 (4)	C2—C1—I1	119.2 (4)
C7—C8—C13	121.6 (4)	C14—C13—C12	118.4 (5)
C9—O1—H1	109.5	C14—C13—C8	123.1 (4)
O1—C9—C10	117.4 (5)	C12—C13—C8	118.4 (5)
O1—C9—C8	122.4 (5)	C16—C17—C12	121.5 (6)
C10—C9—C8	120.2 (5)	C16—C17—H17	119.2
C10—C11—C12	122.2 (5)	C12—C17—H17	119.2
C10—C11—H11	118.9	C15—C14—C13	120.3 (5)
C12—C11—H11	118.9	C15—C14—H14	119.9
C7—N1—C5	122.3 (4)	C13—C14—H14	119.9
C3—C4—C5	120.9 (5)	C15—C16—C17	119.3 (6)
C3—C4—H4	119.6	C15—C16—H16	120.4
C5—C4—H4	119.6	C17—C16—H16	120.4
C1—C6—C5	120.3 (4)	C11—C10—C9	120.7 (5)
C1—C6—H6	119.8	C11—C10—H10	119.7
C5—C6—H6	119.8	C9—C10—H10	119.7
N1—C7—C8	123.1 (4)	C17—C12—C13	118.8 (6)
N1—C7—H7	118.5	C17—C12—C11	121.9 (5)
C8—C7—H7	118.5	C13—C12—C11	119.4 (5)
C2—C3—C4	120.9 (5)	C16—C15—C14	121.7 (6)
C2—C3—H3	119.6	C16—C15—H15	119.1
C4—C3—H3	119.6	C14—C15—H15	119.1
C3—C2—C1	118.3 (5)		
			
C7—C8—C9—O1	−0.8 (7)	C7—C8—C13—C14	1.2 (7)
C13—C8—C9—O1	178.7 (4)	C9—C8—C13—C12	1.1 (6)
C7—C8—C9—C10	178.8 (4)	C7—C8—C13—C12	−179.4 (4)
C13—C8—C9—C10	−1.7 (7)	C12—C13—C14—C15	0.2 (8)
C6—C5—N1—C7	16.5 (7)	C8—C13—C14—C15	179.6 (5)
C4—C5—N1—C7	−164.2 (4)	C12—C17—C16—C15	−0.2 (9)
C6—C5—C4—C3	0.9 (7)	C12—C11—C10—C9	−1.1 (9)
N1—C5—C4—C3	−178.5 (4)	O1—C9—C10—C11	−178.7 (5)
C4—C5—C6—C1	−0.6 (7)	C8—C9—C10—C11	1.7 (8)
N1—C5—C6—C1	178.7 (4)	C16—C17—C12—C13	0.3 (8)
C5—N1—C7—C8	−179.0 (4)	C16—C17—C12—C11	−178.9 (5)
C9—C8—C7—N1	2.2 (7)	C14—C13—C12—C17	−0.3 (7)
C13—C8—C7—N1	−177.2 (4)	C8—C13—C12—C17	−179.8 (4)
C5—C4—C3—C2	−0.4 (8)	C14—C13—C12—C11	179.0 (5)
C4—C3—C2—C1	−0.3 (8)	C8—C13—C12—C11	−0.5 (7)
C5—C6—C1—C2	−0.1 (7)	C10—C11—C12—C17	179.7 (5)
C5—C6—C1—I1	−178.5 (3)	C10—C11—C12—C13	0.5 (8)
C3—C2—C1—C6	0.6 (8)	C17—C16—C15—C14	0.1 (9)
C3—C2—C1—I1	179.0 (4)	C13—C14—C15—C16	−0.1 (9)
C9—C8—C13—C14	−178.3 (4)		

### Hydrogen-bond geometry (Å, º)

**Table:**

*D*—H···*A*	*D*—H	H···*A*	*D*···*A*	*D*—H···*A*
O1—H1···N1	0.82	1.82	2.555 (6)	148
